# Calcium-sensing receptor (CaSR) promotes development of bone metastasis in renal cell carcinoma

**DOI:** 10.18632/oncotarget.24607

**Published:** 2018-03-02

**Authors:** Sebastian Frees, Ines Breuksch, Tobias Haber, Heide-Katharina Bauer, Claudia Chavez-Munoz, Peter Raven, Igor Moskalev, Ninadh D´Costa, Zheng Tan, Mads Daugaard, Joachim W. Thüroff, Axel Haferkamp, Dirk Prawitt, Alan So, Walburgis Brenner

**Affiliations:** ^1^ Department of Urologic Sciences, University of British Columbia, Vancouver Prostate Centre, British Columbia, Canada; ^2^ Department of Urology, Johannes Gutenberg University Medical Center, Mainz, Germany; ^3^ Department of Gynecology, Johannes Gutenberg University Medical Center, Mainz, Germany; ^4^ Current address: Department of Urology, University Clinic Mannheim, Mannheim, Germany; ^5^ Department of Pediatrics, Johannes Gutenberg University Medical Center, Mainz, Germany

**Keywords:** renal cell carcinoma, calcium-sensing receptor, bone metastases, metastasis, kidney cancer

## Abstract

Bone metastasis is an important prognostic factor in renal cell carcinoma (RCC). The calcium-sensing receptor (CaSR) has been associated with bone metastasis in several different malignancies. We analyzed the impact of CaSR in bone metastasis in RCC *in vitro* and *in vivo*. The RCC cell line 786-O was stably transfected with the *CaSR* gene and treated with calcium alone or in combination with the CaSR antagonist NPS2143. Afterwards migration, adhesion, proliferation and prominent signaling molecules were analyzed. Calcium treated *CaSR*-transfected 768-O cells showed an increased adhesion to endothelial cells and the extracellular matrix components fibronectin and collagen I, but not to collagen IV. The chemotactic cell migration and proliferation was also induced by calcium. The activity of SHC, AKT, ERK, P90RSK and JNK were enhanced after calcium treatment of *CaSR*-transfected cells. These effects were abolished by NPS2143. Development of bone metastasis was evaluated *in vivo* in a mouse model. Intracardiac injection of CaSR-transfected 768-O cells showed an increased rate of bone metastasis. The results indicate CaSR as an important component in the mechanism of bone metastasis in RCC. Therefore, targeting CaSR might be beneficial in patients with bone metastatic RCC with a high CaSR expression.

## INTRODUCTION

Renal cell carcinoma (RCC) is among the ten most common cancer sites in men and women. Current estimates show that 62700 new cases will occur in the United States in 2016 and 14420 patients will die of their disease [1]. Widespread usage of cross-sectional imaging and ultrasound for other medical conditions have led to a shift towards detection of earlier stages of RCC [2, 3]. Nevertheless, about 10% of all patients diagnosed with RCC have metastasis at presentation and another 20–30% of patients will develop metastasis despite an initially curative treatment approach [4].

The most common sites of metastasis in RCC include lung (60%), bone (35%) liver (20%) and brain (10%) [5, 6]. Bone metastasis include rib and pelvis (50%) as well as spine (40%), often leading to significant pain in patients and decrease in quality of life [7]. Therefore, limiting bone metastasis in patients with advanced renal cell carcinoma is a crucial treatment goal.

The calcium-sensing receptor (CaSR), a G-protein coupled receptor, is involved in the normal calcium and phosphate homeostasis, renin release as well as in the acidification and concentration of urine [8–10] and therefore highly expressed in normal kidney tissue [11]. With an increase in extracellular calcium, CaSR respond with an activation of several intracellular signaling pathways, leading to changes in cell proliferation, apoptosis and migration [12]. In other cancers such as breast and prostate cancer, the expression of CaSR has been identified to correlate with an increased affinity of the cancer towards development of bone metastasis [13, 14]. Our group has recently identified the same finding in a subset of patients with metastatic RCC [15]. Objective of this study was to evaluate the effect of CaSR overexpression on the development of bone metastasis *in vitro* and *in vivo*.

## RESULTS

### CaSR transfection of RCC cells was successful

To evaluate the success of CaSR gene transfection in the 786-O cells, PCR, Western blot and flow cytometry analyses were performed. The CaSR-transfected cell line showed a much higher CaSR expression than vector-transfected control cells (Supplementary Figure 1). Therefore, these cells were selected for the functional assays.

### Calcium induced an increase in cell adhesion, migration and proliferation of CaSR-transfected 786-O cells *in vitro*

**Figure 1:**
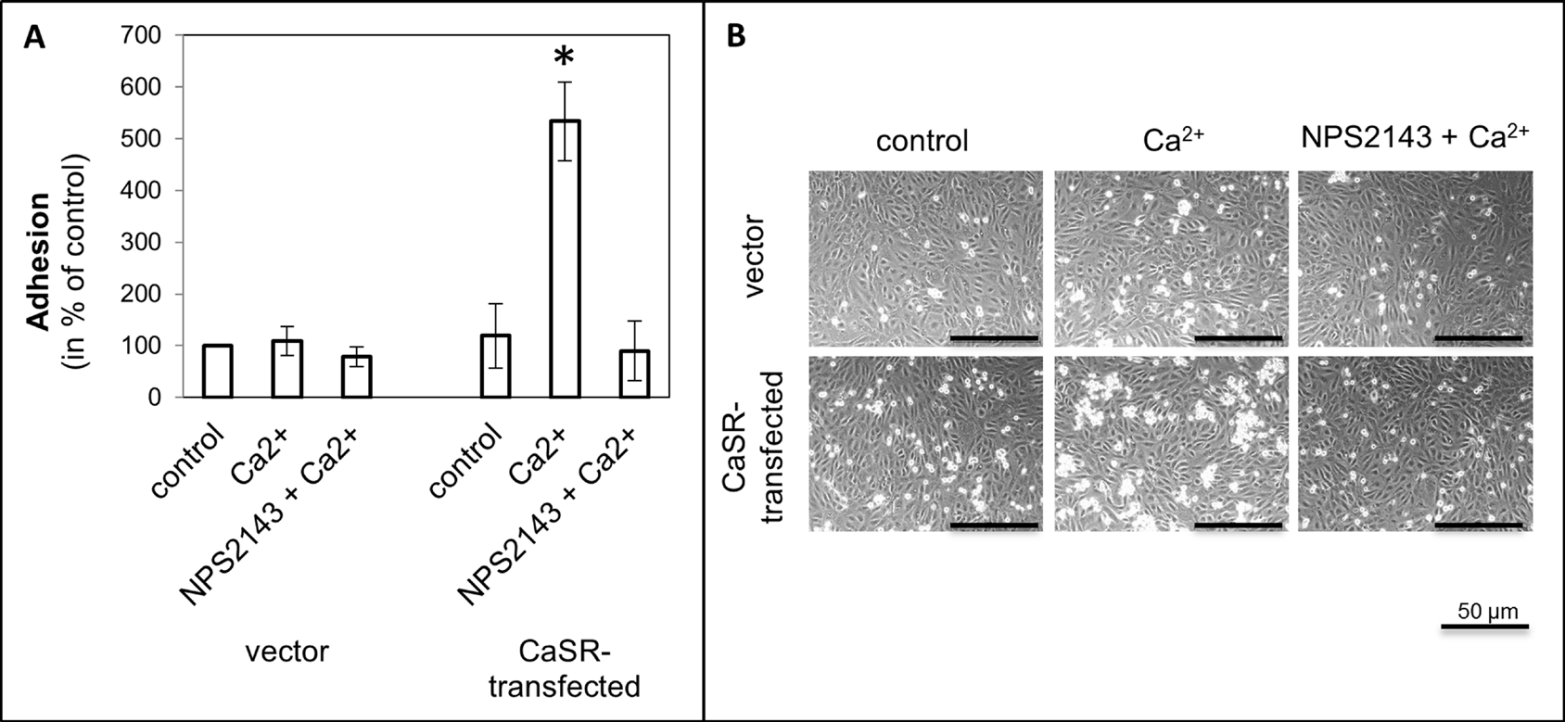
Cell adhesion of CaSR-transfected 786-O cells on endothelial cells (HUVEC) Cells were treated with calcium (5 mM) or a combination of calcium (5 mM) and NPS2143 (10 µM). (**A**) The adhesion value is shown as percentage of the adhesion of untreated vector-transfected cells. (**B**) Microscopic images of cell adhesion on HUVEC. Calcium triggered cell adhesion on HUVEC in CaSR-transfected cells significantly. Significance was calculated by Student’s *T*-test, *p <* 0.05.

**Figure 2:**
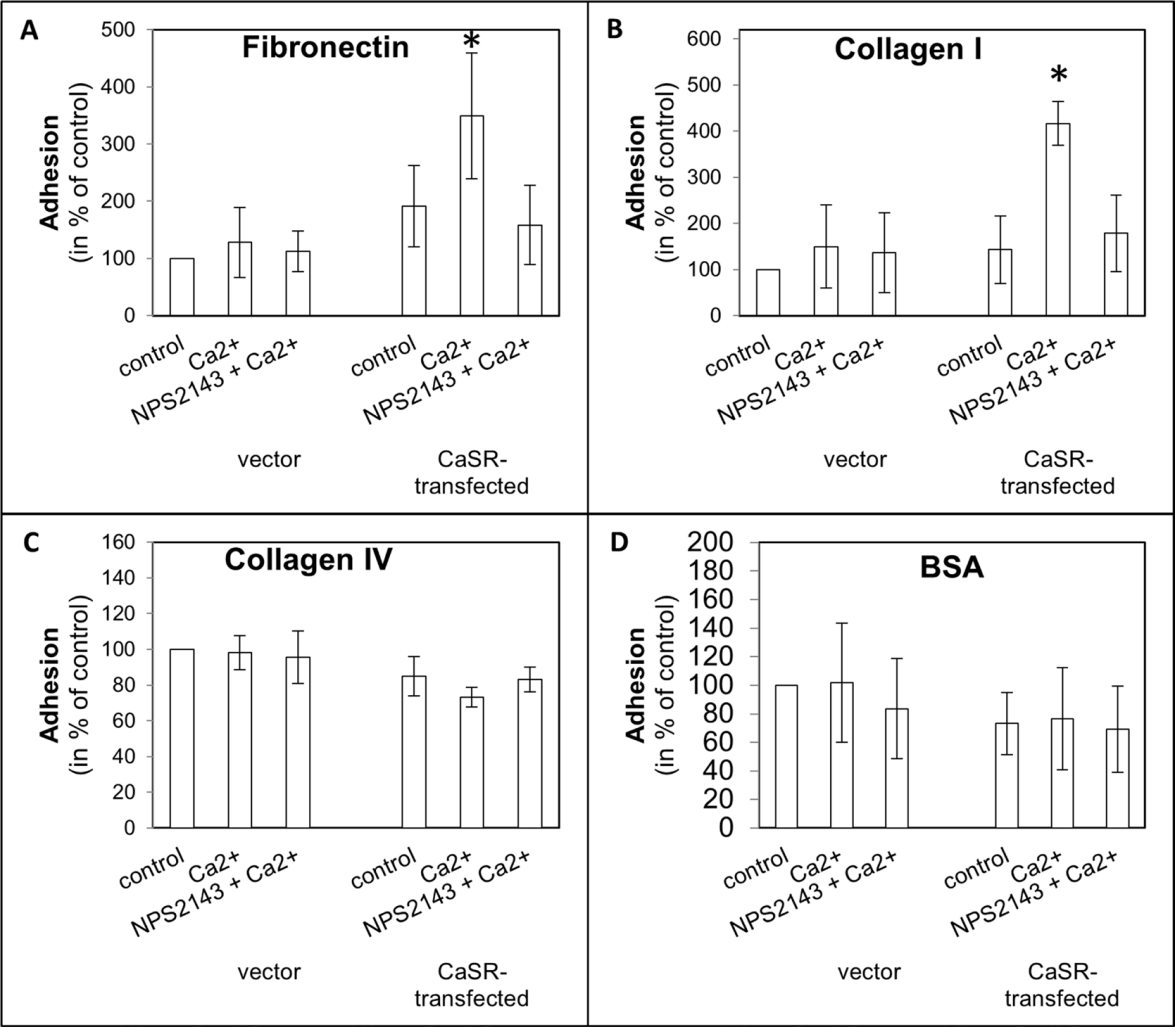
Cell adhesion of CaSR-transfected 786-O cells on extracellular matrix components fibronectin (**A**), collagen I (**B**), collagen IV (**C**) and BSA (**D**). Cells were treated with calcium (5 mM) or a combination of calcium (5 mM) and NPS2143 (10 µM). The adhesion value is shown as percentage of the adhesion of untreated vector-transfected cells. BSA was used as control. Calcium triggered cell adhesion on fibronectin and collagen I in CaSR-transfected cells significantly. Significance was calculated by Student’s *T*-test, *p <* 0.05.

**Figure 3:**
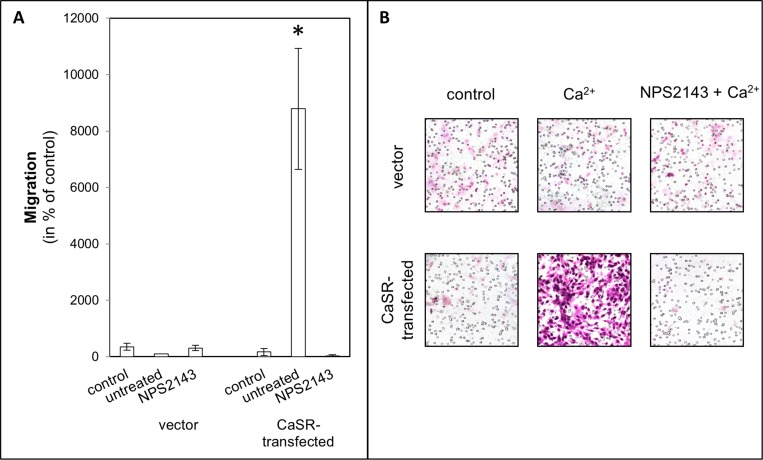
Chemotactical cell migration of CaSR-transfected 786-O cells using calcium as chemotaxin Cells were treated with NPS2143 (10 µM). Migration was determined in a Boyden chamber using serum-free medium as control or calcium (5 mM) as chemotaxin. (**A**) The migration value is shown as percentage of the migration of untreated vector-transfected cells. (**B**) Microscopic images of migrated cells. CaSR-transfected cells showed a significant increased migration. Significance was calculated by Student’s *T*-test, *p <* 0.05.

**Figure 4:**
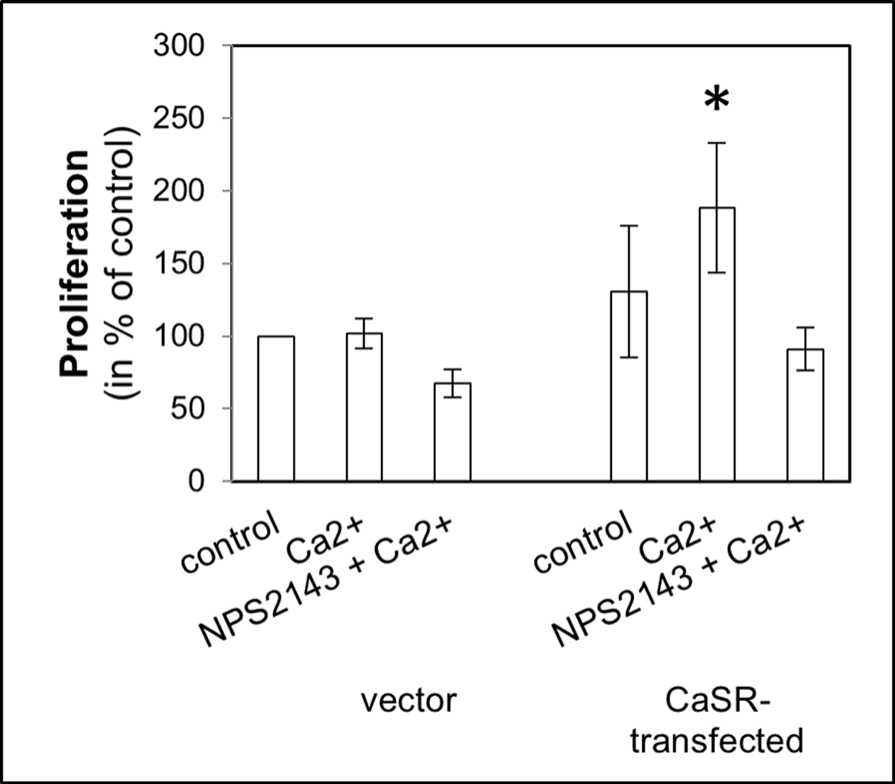
Cell proliferation of CaSR-transfected 786-O Cells were treated with calcium (5 mM) or a combination of calcium (5 mM) and NPS2143 (10 µM). The proliferation value is shown as percentage of the proliferation of untreated vector-transfected cells. Calcium triggered cell proliferation in CaSR-transfected cells significantly. Significance was calculated by Student’s *T*-test, *p <* 0.05.

To evaluate the steps of metastasis, cell adhesion on endothelial cells, adhesion on ECM compounds, migration with calcium as chemoattractant was used and cell proliferation was measured, depending on calcium treatment. The use of calcium as a CaSR activator led to a significant increase of cell adhesion to endothelial cells (5.3 ± SEM or SD-fold, *p <* 0.0001, Figure 1) as well as adhesion to fibronectin (3.5-fold, *p =* 0.017, Figure 2A) and collagen type I (4.2-fold, *p <* 0.001, Figure 2B) in the CaSR transfected 786-O cells. The adhesion to collagen type IV (Figure 2C) and to the BSA negative control were unchanged (Figure 2D). The chemotactical cell migration using calcium as a chemoattractant (Figure 3) and cell proliferation (Figure 4) were also significantly enhanced in CaSR transfected 786-O cells, 88-fold (*p =* 0.002) and 1.9-fold (*p =* 0.027), respectively. By the combinatory use of calcium and NPS2143, a specific CaSR inhibitor, the observed effects of the calcium treatment were reversed nearly down to normal activities (Figures 1–4). The optimal concentration of 10 µM NPS2143 was determined using a MTT-based cell viability assay (Supplementary Figure 2).

### CaSR activation induced enhanced MAPK and AKT signaling

**Figure 5:**
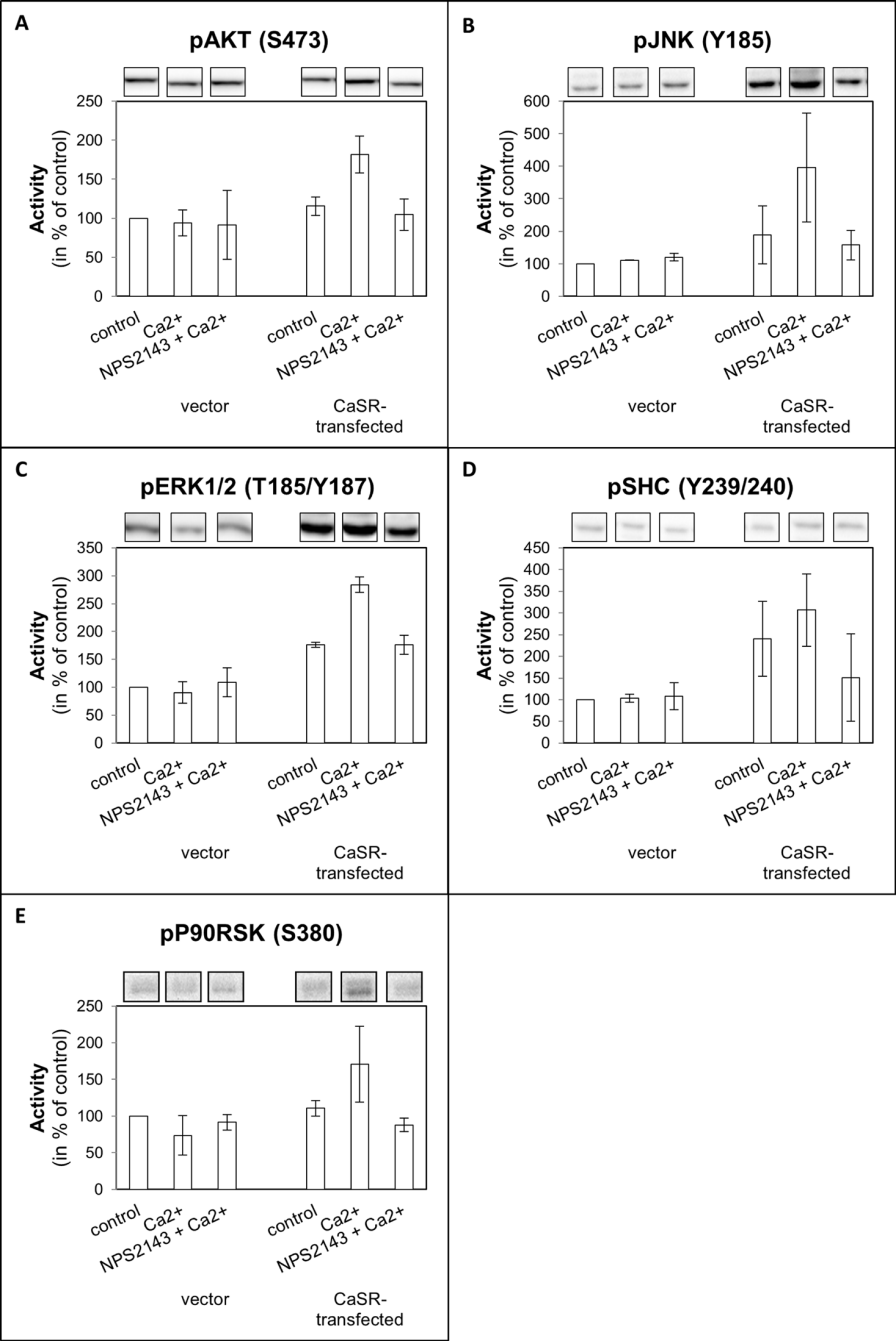
Activity of (**A**) AKT, (**B**) JNK, (**C**) ERK1/2, (**D**) SHC, and (**E**) P90RSK of CaSR-transfected 786-O. Cells were treated with calcium (5 mM) or a combination of calcium (5 mM) and NPS2143 (10 µM). The activity value is shown as percentage of untreated vector-transfected cells. Exemplary Western blot bands are shown above the diagram. Calcium triggered activity of AKT, JNK, ERK1/2, SHC and P90RSK in CaSR-transfected cells.

To get an overview about the effect of calcium on the activation of intracellular signaling pathways a human phospho-kinase array was accomplished using CaSR-transfected 786-O cells. Those signal transduction mediators which were sensitive for calcium in CaSR-transfected cells but not in control cells (Supplementary Figure 3) were verified by Western blot analysis. In 786-O cells the AKT and MAPK signaling pathways were activated by calcium in CaSR-transfected, but not in vector-transfected cells. Activation of CaSR resulted in enhanced phosphorylation of the CaSR downstream targets SHC, AKT, ERK, JNK and p90RSK. These effects were abolished by the CaSR antagonist NPS2143 (Figure 5).

### Overexpression of CaSR led to a higher rate of bone metastasis *in vivo*

**Figure 6:**
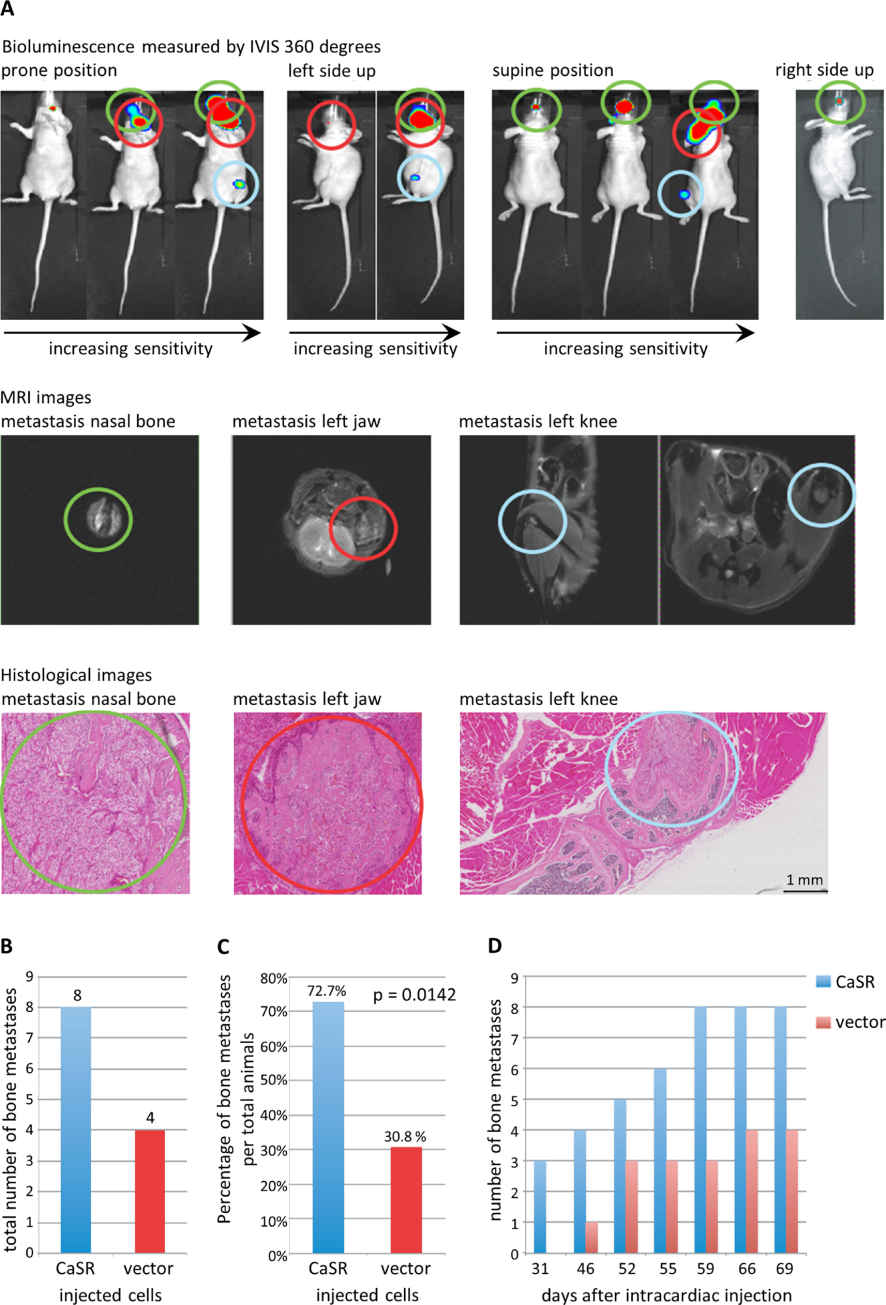
Development of bone metastases after intracardiac injection of CaSR overexpressing cells into a xenograft mouse model Detection of bone metastases using bioluminescence (IVIS), representative MRI-images and histopathology (**A**) (representative images shown - each single lesion is represented by one color) confirmed a higher number of total bone metastases (**B**) as well as a higher number of bone metastases per total animals (**C**) (Students *T*-Test, *p <* 0.05) and an earlier development of bone metastases (**D**) for the CaSR overexpressing cells.

After intracardiac injection of the CaSR and luciferase transfected 786-O cells in mice, the first metastases were detected after 31 days. Location of the metastasis was precisely determined by initial detection with bioluminescence, followed by MRI in representative cases and final confirmation with histopathology (Figure 6A). At the end of the experiment (day 69), we detected 16 metastases in 11 mice, 75% of which were located in bones. In our xenograft model, overexpression of CaSR in the 768-O cells led to a significant increase in bone metastases compared to vector-transfected 768-O cells. We saw a total of 8 bone metastases in CaSR group and 4 bone metastases in the vector group (Figure 6B). The percentage of metastases per total number of mice was 72.73% in the CaSR and 30.77% in the vector group (*p =* 0.0142) (Figure 6C). Mice injected with CaSR overexpressing cells showed the first bone metastasis earlier than mice injected with control cells (Figure 6D). In total 8 of 24 injected mice (25%) had relevant bone metastasis. Table 1 shows the frequency of the metastatic distribution, with 43.75% located in the jaw of the animals.

**Table 1:** Frequencies of relevant metastatic locations after 69 days

Location of metastasis	CaSR overexpressing cells	Vector	Total
Spine	2 (22.2%)	0	2 (12.5%)
Jaw	4 (44.4%)	3 (42.86%)	7 (43.75%)
Extremities	1 (11.1%)	0	1 (6.25%)
Pelvis	1 (11.1%)	1 (14.29%)	2 (12.5%)
Muscle	1 (11.1%)	1 (14.29%)	2 (12.5%)
Heart	0	2 (28.57%)	2 (12.5%)
Total number of metastasis	9 (100%)	7 (100%)	16 (100%)
Total number of bone metastasis	8 (66%)	4 (33%)	12 (100%)
Total number of animals	11	13	24

## DISCUSSION

Cancer research has been elucidating mechanisms of metastasis for decades. The explanation of the preference of cancer cell colonies to specific metastatic sites is not fully understood. However, the interaction of the cancer cells of the initial tumor with the microenvironment of the site of metastasis seems of great importance.

During bone metastasis cells invade the calcium rich bone structure and increase the bone turnover by interacting with osteoclasts and osteoblasts, resulting in high levels of extracellular calcium, numerous growth factors and cytokines in the tumor microenvironment [16]. Cancer cells metastasizing to the bone may benefit from this unique condition by increased attraction of cancer cells as well as facilitation of metastatic seeding and proliferation. In contrast, treatments such as bisphosphonates or RANK-ligand (RANKL) inhibitors have shown to change this environment by inhibition of osteoclast activity and osteoclastogenesis leading to tumor cell apoptosis [17, 18].

The calcium-sensing receptor plays an important role in the recognition of calcium levels [19]. It is not only responsible for maintaining calcium hemostasis by regulation of the parathyroid hormone, but also appears to play a crucial role in the development of bone metastases in several cancers such as breast and prostate cancer [13, 20–22]. Chirgwin *et al.* proposed a kind of vicious circle where the tumor cells secrete parathyroid hormone-related protein (PTHrP) after calcium stimulation, which results in an increased RANKL expression on immature osteoblasts. This in turn activates osteoclastogenesis via RANK on osteoclast precursors, leading to an increase in osteolysis and consequently to increasing calcium concentration in the tissue, again stimulating tumor cells. Furthermore, growth factors and cytokines like TGFβ, PDGF and IGF1 are released during osteolysis, which have a proliferation inducing effect on tumor cells and additionally promote bone metastasis progression [23, 24].

Boudot *et al.* recently showed that a CaSR overexpression could be linked to an increase of the osteolytic potential of breast cancer cells [25]. In prostate cancer a similar mechanism with involvement of prostate specific antigen (PSA) and endothelin-1 (ET-1) has been described [26].

As the kidney plays a crucial role in calcium homeostasis in healthy tissue, CaSR is widely expressed in normal and malignant renal tissues [11]. We recently demonstrated that the expression of CaSR was highest in specimens and primary cells of patients with renal cell carcinoma who developed bone metastases in a period of five years after surgery [15]. Based on this observation we generated the hypothesis that overexpression of CaSR may result in an increase of cellular processes, which trigger bone metastasis. It is a well-established concept that due to their osteolytic characteristics, RCC bone metastases lead to high calcium levels in serum [7], potentially inducing enhanced cellular activity of CaSR-expressing tumor cells. In fact, we here show that overexpression of CaSR induced calcium-dependent a higher adhesion, migration and proliferation potential in RCC cell line 786-O *in vitro,* suggesting that enhanced CaSR expression in RCC cells results in increased bone metastasis.

Metastatic progression is a multistep process including several cellular processes. The first step of metastatic progression is the adhesion of the tumor cells on the endothelium. In bone metastases, tumor cells are attracted to the bone by various extracellular components and also by free calcium ions. The adhesion potential on the extracellular matrix differed in our study depending on the compound. The adhesion potential to fibronectin and collagen type I but not to collagen type IV was increased. Collagen I is the main component of the organic part of the bone, and fibronectin is also highly concentrated in bone tissue [27], so that both seem to chemoattract RCC cells. In contrast, collagen IV is mainly found in the basal lamina of blood vessels, less developed in bone due to the fenestrated character of the endothelium [28]. This supports the hypothesis that expression of CaSR guides the tumor cells to the bone and emphasized the organ-specific metastasis of tumor cells. The CaSR inhibitor NPS2143 revised the described cellular effects, demonstrating its CaSR dependence. These results show that in our cell system calcium, via CaSR, induces enhanced metastatic behavior of RCC cells leading to the development of bone metastasis. A contribution of the calcium-sensing receptor to cell adhesion and migration via integrin signaling was already shown by Tharmalingam *et al.* in medullary thyroid carcinoma cells [29]. Tumor cells harboring the calcium-sensing receptor could enhance these cellular processes downstream via the integrin signaling cascade and also via the CaSR signaling cascade itself. Downstream targets are primarily the AKT and MAPK signaling cascade. We analyzed intracellular signaling pathways involved in the CaSR dependent metastatic behavior of the RCC cells and found a significance of MAPK and AKT signaling cascades. Kinase activity of the MAPK, JNK, ERK1/2 and p90RSK as well as the kinase AKT was enhanced after stimulating CaSR-transfected tumor cells with calcium. An enhanced activity was also found for the upstream adapter protein SHC. As already shown in our functional cell analyses, as documented by using the CaSR inhibitor NPS2143, the effect of cell signaling were CaSR dependent. A contribution of the MAPK and AKT signaling pathways in these processes was already shown in breast cancer cells by Saidak *et al.* and in primary RCC cells in our former investigation [15, 30]. In prostate cancer an increase in skeletal metastasis has been associated with AKT signaling [20].

The CaSR seems to induce metastasis by activating adhesion and migration. Besides these processes, another important step of metastatic progression is the maintenance of cell proliferation. Tumor cells reaching the bone microenvironment are exposed to high calcium concentrations, which in return activate the receptor. It has been shown that CaSR is able to trigger cell proliferation in smooth muscle cells as well as in pancreatic β-cells [31, 32]. Our results support the hypothesis that CaSR guides the tumor cells to the bone and the development of organ-specific metastasis of tumor cells. Therefore, the compatibility between the tumor cells (“the seed”) and the microenvironment ("the soil") has to fit for a successful metastatic spread [33].

We confirmed our *in vitro* results by intracardiac cell injections in a xenograft mouse model *in vivo*. Detection of metastasis is highly sensitive in this animal model and has the great benefit of enabling a continuous monitoring [34]. Mice injected with CaSR-transfected tumor cells showed a higher rate of bone metastases in total as well as an earlier occurrence of metastases compared to mice that were injected with vector-transfected cells.

**Figure 7:**
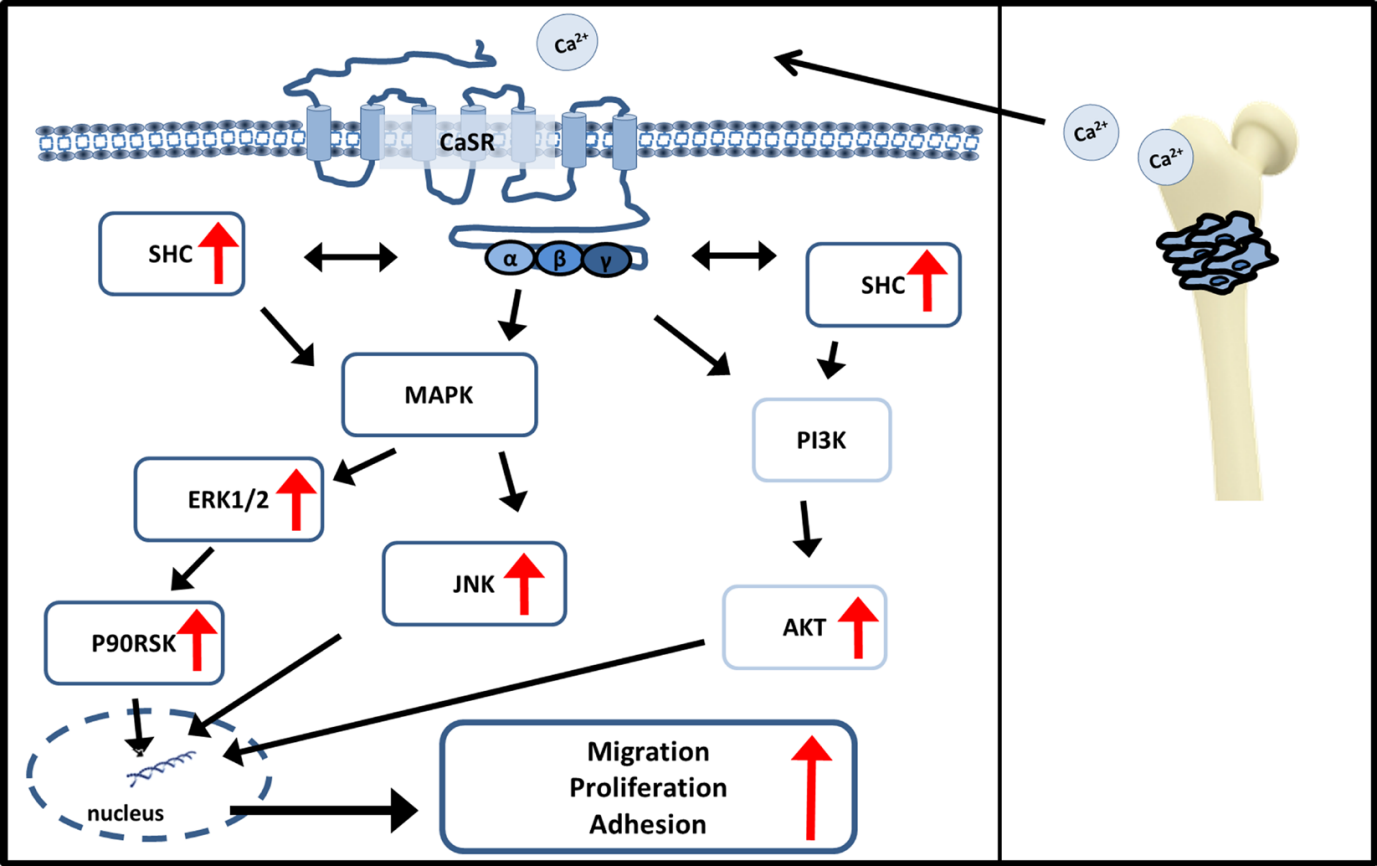
Signaling pathways influenced by CaSR CaSR, stimulated by calcium, triggers AKT activity as well as MAPK activity of JNK, ERK1/2, p90RSK and SHC activity. Both signaling pathways lead downstream to an increase in cellular processes like cell migration, proliferation and adhesion. Red arrows show increased activity in CaSR overexpressing cells after Calcium stimulation.

This indicates a contribution of AKT and MAPK pathways after calcium dependent stimulation of CaSR in triggering cell migration, adhesion and proliferation, thus enhancing bone metastasis (Figure 7). Our results propose CaSR as a potent target preventing bone metastasis in renal cancer. NPS2143, a calcilytic drug [35], may serve as a new therapeutic agent. Marquis *et al.* showed that NPS2143 had a good specificity with only a few additional targets like monoamine transporters [36], which may lead in minor side effects. It was recently tested for osteoporosis treatment and calcium hemeostasis related diseases [37–39], and *in vivo* has demonstrated a good biocompatibility [40, 41]. Thus, RCC patients with bone metastases and increased CaSR expression might benefit from a therapy with antagonists like NPS2143, justifying further preclinical and clinical development.

## CONCLUSIONS

Our results identify CaSR as an important promoter of bone metastasis in RCC. The receptor is responsible for a calcium-dependent increase in cell migration, adhesion and proliferation resulting in an increase of bone metastases. Therefore, targeting CaSR may be beneficial in patients with bone metastases of RCC with high CaSR expression and development of CaSR as a therapeutic target is warranted.

## MATERIALS AND METHODS

### Cells, cell culture and treatment

Human renal cell carcinoma cell line 768-O was obtained from LGC Promochem (Wesel, Germany). Cells were cultured in AmnioMAX C100 Basal Medium supplemented with AmnioMAX C-100 Supplement (Gibco). The cell line was incubated in a moistened atmosphere at 5% CO_2_ at 37° C. Every time cells were passaged they were washed with sterile DPBS, and detached by using 2.5% trypsin-EDTA. Cells were centrifuged at 300 g for five minutes and resuspended in culture medium. For the experiments, cells were treated with calcium alone (5 mM, 30 min, serum-free) or alternatively with the CaSR antagonist NPS2143 (10 µM, 30 min, serum-free) and afterwards with the combination of NPS2143 (10 µM) and calcium (5 mM) for further 30 min. Serum-free medium served as a control.

### CaSR transfection

The human *CaSR* gene (full length) was cloned into a pcDNA3 vector system (Invitrogen) containing a gentamycin selection gene. The plasmid was prepared by using the Qiagen plasmid maxi kit (Qiagen). 786-O cells were stably transfected using Turbofect transfection reagent (Thermo Scientific, USA). 786-O cells were seeded at 2 × 10^5^ cells/well in a 6-well-plate in serum-free medium and incubated overnight at 37° C. Next, 4 µg of plasmid DNA (CaSR) were diluted in 400 µl serum-free medium, immediately mixed with 6 µl of Turbofect each and incubated for 20 min at room temperature for complex formation. 400 µl of transfection mixture were carefully suspended into each well of the 6-well-plate. After 36 h the medium was changed to the selection medium containing 400 mg/ml gentamycin. As negative controls we used the empty vector. The effectiveness of the CaSR overexpression was determined using flow cytometry, PCR and Western blot analyses.

### Luciferase transfection for *in vivo* experiments

For *in vivo* experiments CaSR transfected (786-O-CaSR) or empty vector transfected 768-O cells (786-O-vector) were additionally infected with a lentivirus containing the firefly luciferase gene as previously described [42]. Briefly, 10 µg of the transducing vector, FUGWB plasmid was mixed with 10 µg of packaging vector pR8.91 and 5 µg of plasmid encoding vesicular stomatitis virus glycoprotein(VSV-G) with the addition of 37 µl of CaCl_2_ (2 M, pH 7.2). The DNA–calcium complex was mixed with 250 µl of 2x HEPES buffered solution and after 30 min incubation was added to 293T cells. After 24 h the medium was replaced and after additional 24 h the supernatant was collected, filtered through a 0.45 µm filter and used to infect the 768-O cells. Blasticidin at a concentration of 5 µM was used to select the transduced cells. A PCR was performed after the transfection of both plasmids to confirm the overexpression of CaSR in the 768-O cells. In addition the cells were tested *in vitro* for luciferase activity (data not shown).

### Flow cytometry

Quantification of CaSR expression was performed by flow cytometry as described previously [15]. The cells (2 × 10^6^ cells/ml) were fixed using 4% paraformaldehyde for 10 min. Mouse monoclonal anti-CaSR antibody (Novus Biologicals, USA) was used in a 1:1000 dilution. Mouse anti-human isotype control immunoglobulins (Dako, Carpinteria, USA) was used in the same concentration in PBS containing 1% bovine serum albumin (BSA) for 60 min at 4° C. After washing with PBS the secondary antibody (alexa flour 488 goat anti-mouse) was diluted 1:1000 in 1% BSA/PBS and incubated for 20 min at 4° C in darkness. Cells were washed again and CaSR expression was quantified in a flow cytometer (BD Calibur, Becton Dickinson, Heidelberg, Germany).

### Cell adhesion to the endothelial cells

Human umbilical vein endothelial cells (HUVEC, 5 × 10^5^ cells/well) were cultivated on 6-well-plates, previously coated with 0.2% gelatin solution for 30 min at 37° C, in a CO_2_ incubator to a confluent monolayer. 5 × 10^5^ of transfected 786-O cells were transferred into the 6-well-plates for 2 h at 37° C in a humidified atmosphere containing 5% CO_2_ in air. Cells were washed with DPBS and fixed for 10 min with 1% glutaraldehyde. Quantification was performed by counting adherent cells, which could be distinguished from HUVEC on the basis of their shape. The adherent cells were counted in five different fields with an area of 156, 35 mm^2^ each, using a raster ocular.

### Cell adhesion to ECM compounds

Cell adhesion assay to ECM compound was performed as described previously [43], using a Reacti-Bind™ amine binding 96-well-plate, coated with fibronectin, collagen I and IV or BSA (all 10 µg/ml) as control over night. On the next day, wells were washed twice with DPBS with 0.05% Tween 20. Unspecific binding sites were blocked with 200 µl blocking solution (DPBS with 0.5% BSA) per well and incubated for one hour in a moistened atmosphere at 5% CO_2_ at 37° C in air. Meanwhile cells were detached with trypsin-EDTA and treated for 30 minutes with calcium and/or NPS2143, respectively. Blocking solution was removed and 50 µl of tumor cell suspension (4 × 10^5^ cells/ml) per well were added. After one hour incubation in a moistened atmosphere at 5% CO_2_ and 37° C, non-adherent cells were washed out with 2 × 200 µl washing buffer per well. Adherent cells were fixed with 100 µl 4% paraformaldehyde (Histofix 4%, Roth) per well for 15 minutes at room temperature. Adherent and fixed cells were stained using 100 µl crystal violet solution (5 mg/ml in 2% ethanol) for 10 minutes at room temperature. Afterwards the staining solution was washed out and the plate was air-dried. For resolving the colorant wells were incubated with 100 µl 2% SDS (Roth) for 30 minutes on a rocking shaker. The absorbance was measured at 550 nm (reference value at 650 nm).

### Cell migration assay

Cell migration was quantified in a microchemotaxis chamber (Boyden chamber, Costar, Cambridge, USA) including a porous polycarbonate membrane (pore diameter 8 μm; Neuroprobe Inc., Gaithersburg, USA) with a surface of approximately 7.8 mm^2^ per well [44]. Before analysis cells were cultivated in serum-free culture medium for 24 hours. The lower part of the chamber was filled with 29 μl (manufacturer’s instruction) calcium (10 mM, according to the Calcium concentration in bone [45]) in serum-free medium or medium alone as control. The upper chamber part was loaded with 50 μl of the tumor cell suspension (3 × 10^5^ cells/ml) followed by an incubation step of 16 h at 37° C in a humidified atmosphere containing 5% CO_2_ in air. Cells that did not pass the polycarbonate membrane were removed from the upper side of the porous membrane by washing with a Weise buffer (Merck, Darmstadt, Germany; a potassium dihydrogen phosphate buffer, pH 7.0) and by mechanic removal using a cell scraper. Membranes were dried and fixed in methanol for 1 min followed by immediately staining with hemacolor (Merck). The dyed membrane was transferred to a microscope slide and covered with immersion oil. Cell migration capability was evaluated thereafter (Zeiss, 400-fold magnification). For a single determination, ten different views per well with a combined membrane surface of 2.5 mm^2^ were evaluated. Experiments were performed in quadruplicates. From the results a mean value and a standard error were calculated [46].

### Cell proliferation assay

Cell proliferation experiments were performed using a colorimetric BrdU incorporation assay (Roche, Mannheim, Germany) [47].Transfected cells were seeded on a 96-well-plate (5 × 10^3^ cells/well), cultured for 48 h and treated in quadruplicates with calcium or calcium and NPS2143, as described. BrdU solution was added to the cells without replacing the NPS2143 and/or calcium containing culture medium and incubated for 2 h in presence of calcium at 37° C in a CO_2_ incubator. Tumor cells were fixed and DNA was denatured in one step by adding fixDenat™ solution for 30 min. Incorporated BrdU was detected by an anti-BrdU-POD antibody within 60 min. The resulting immune complex was detected by a subsequent substrate reaction and quantified by measuring the absorbance at 450 nm (reference wavelength 690 nm).

### MTT assay

Cell viability after treatment with NPS2143 was analyzed using MTT assay (3,(4,5-Dimethylthiazol-2 yl)-2,5-Diphenyl-Tetrazoliumbromid). Cells were seeded on a 96-well-plate (5 × 10^3^ cells/well), cultured for 24 h and treated in quadruplicates with increasing concentrations of NPS2143 in a range from 100 nM to 100 µM. After 24 h 20 µl of MTT (0.5%) was added. After an incubation of 3 h at 37° C in 5% CO_2_ atmosphere, cells were washed with 200 µl DBPS. The wells were incubated with 100 µl isopropyl alcohol for 15 min and absorbance at 570 nm was measured [48].

### Human phospho-kinase array

The activity of 45 intracellular signaling kinases was quantified by using a human phospho-kinase array (R&D, Minneapolis, USA). Protein extracts from CaSR or empty vector transfected 786-O cells, were prepared by using 200 μl lysis buffer included in the kit. Cells were scraped off with a cell scraper in 400 µl of lysis buffer of the kit. All further steps were performed in accordance with the instructions in the manual. Protein concentrations were determined using BCA (bicinchoninic acid) reagent (Thermo Scientific, Rockford, USA). For the analysis 300 µg protein was used. The provided membranes were incubated with array buffer 1 for 1 h on a rocking platform. On each membrane 1 ml of the protein lysates (300 μg) were added and incubated overnight at 4° C on a rocking platform. After a triple washing step (washing buffer) membranes were incubated in antibody cocktails for 2 h on a rocking platform. After a 30 min treatment with streptavidin-HRP solution, membranes were exposed to a chemiluminescent reagent. Positive signals were visualized using a chemiluminescence imaging system. The amount of protein in each spot was calculated by using image software provided by the chemiluminescence Imaging System.

### Western blot analysis

For preparation of protein extracts, 786-O cells were cultivated on 10 cm sterile dishes. Reaching semi-confluent growth, cells were cultured in serum free media for 24 h followed by treatment with calcium or a combination of calcium and NP2143 as described above. Afterwards cells were covered with 500 µl cell lysis buffer (2 mM HEPES, pH 7.7, 0.02 M NaCl, 0.05 mM MgCl_2_, 0.04 mM EDTA, 0.1% Triton X-100, 5 µM DTT including 1% protease and phosphatase inhibitors (Sigma, Steinheim, Germany)) /dish. Cells were mechanically removed from the surface using a sterile cell scraper transferred to a tube and immediately vortexed. After 30 min incubation on ice the extracts were centrifuged at 14000 g, 4° C for 10 min. Protein concentrations of the extracts were determined using BCA (bicinchoninic acid) reagent (Thermo Scientific, Rockford, USA). Equal amounts of protein extracts (50 μg per lane) were separated by SDS-PAGE (sodium dodecyl sulfate-polyacrylamide gel electrophoresis) with 10% polyacrylamide gels (Rotiphorese-Gel 30, Roth, Karlsruhe, Germany) and transferred onto PVDF membranes (Poly Screen PVDF Transfer Membrane, Perkin Elmer, Rodgau, Germany) by semi-dry blotting. Membranes were blocked according to instruction manual of antibody manufacturer for one hour. Next, membranes were incubated with primary antibodies in blocking solution (tris buffered saline (TBS), 0.1% Tween 20 and 5% non-fat milk) at 4° C overnight. The antibodies against CaSR (novus biologicals), phospho-AKT (Ser473, Cell Signaling, Boston, USA), phospho-ERK1/2 (ERK1 Thr202/Tyr204, ERK2 Tyr185/Tyr187, R&D Systems, Minneapolis, USA), phospho-JNK (Y185), phospho-SHC (Y239/240) and phospho-P90RSK (S380), all from Cell Signalling, Boston, USA, were diluted 1:1000. After washing the membrane three times it was incubated with HRP-conjugated secondary antibodies (DAKO) at a dilution of 1:1000 for one hour at room temperature. Antigens were visualized by an enhanced chemiluminescence solution (ECL, Perkin Elmer Life Sciences, Waltham, USA) using a chemiluminescence imaging system (Fluorchem E (Biozym)). For quantification a computer-based pixel counting system was used (AlphaView, Protein Simple), subtracting the background from the visual band. These values were normalized to Coomassie stained amounts.

### Tumor xenografts

All animal studies were performed in accordance of the guidelines of the Canadian Council on Animal Care with institutional certifications (University of British Columbia). Under gas anesthesia, 5 × 10^6^ 768-O-CaSR and 768-O-vector cells were injected into the left ventricle of 8- to 10-week-old nude mice via ultrasound guidance (small animal imaging system Vevo 770, FUJIFILM VisualSonics). Each cell line was implanted into 15 mice. Four mice in the CaSR and two mice in the vector group had to be sacrificed immediately after injection. The remaining mice were monitored twice weekly for weight and daily for appearance. All animals were euthanized according to the animal ethics protocol at the completion of the study (day 69).

### Imaging

The establishment of metastases was followed by bioluminescence imaging (IVIS^®^ Lumina; PerkinElmer). Mice were examined by IVIS weekly for 2 weeks and afterwards twice weekly in 360 degrees, by four consecutive images and rotation of the animal that each side faced towards the sensor in one image to reduce confounding by absorption of bioluminescence by tissue covering the metastases. In addition, before euthanasia the animals were injected with luciferin and after confirming bioluminescence activity, rapidly dissected according to our template. The number of hotspots shown in the IVIS images pre and post-mortem showed a complete correlation. Histologic images of each suspected metastasis were performed to identify the exact location.

### MRI Imaging

MRI experiments were completed using a 7 Tesla Bruker Biospec 70/30 scanner to evaluate the accuracy of the IVIS imaging. A volume coil with 70 mm i.d. was used for signal transmission and reception. RARE T2-weighted scans were acquired and used to identify areas with possible bone metastasis, which would appear hyperintense (TE = 46.1 ms, RARE factor = 8, FOV = 9 × 4 cm, 256 × 256, slice thickness = 1.5 mm). Diffusion images were then acquired in these regions using either DTI-EPI or Spin-Echo DTI with a field-of-view of 4 cm × 4 cm, a matrix size of 128 × 128, a slice thickness of 1.5 mm, and one diffusion direction. DTI-EPI was taken using 8-shots, an echo time of 32.84 ms, a 3000 ms repetition time, and 6 averages. Spin-Echo DTI had an echo time of 21.337 ms, a repetition time of 1200 ms and 1 average. We could demonstrate that only metastasis with a bioluminescent response of at least 10^6^ photons/sec could be detected in the MRI. MRI was performed on two animals per group. Therefore, tumors with a bioluminescence of at least 10^6^ photons/sec were classified as relevant metastasis. We did not find any other metastasis on MRI that were not seen on IVIS imaging.

### Tissue extraction

To locate metastasis in our mouse model, animals were injected with luciferin 10 min before euthanasia and 4 IVIS images were taken to screen for metastasis. After euthanasia, the skin of the mice was removed and each animal was dissected using a template. Extremities, lower and upper spine, left part of head, right part of head, tail, thorax, pelvic area and every organ were imaged with IVIS, separated from each other in 6-well-plates. Areas with increased bioluminescence were further dissected to isolate the metastasis from surrounding tissue. Samples were then stored in 4% paraformaldehyde for 24 hours at 4° C and switched to 70% Ethanol thereafter.

### Histology

Before immunohistochemical staining decalcification was performed. Tissues were left in 0.5 molar EDTA on the shaker to facilitate fluid infiltration for a total of 7 days.. EDTA solution was refreshed every 2–3 days. Once decalcification was complete, tissue was washed with distilled water then store in 70 % ethanol prior to tissue processing. Immunohistochemical staining was performed on formalin-fixed, paraffin-embedded 5 μm tumor sections using the appropriate primary antibody (haematoxylin and eosin)) and the Ventana autostainer Discover XT (Ventana Medical Systems).

### Statistical analysis

Differences between the two groups were compared using Student *t* test and Mann–Whitney *U* test. All statistical calculations were performed using IBM-SPSS 24.0 software and Excel 2010 and *P* values < 0.05 were considered significant.

## SUPPLEMENTARY MATERIALS FIGURES



## Abbreviations

AKT: AKT8 virus oncogene cellular homolog
Ca^2+^: calcium
CaSR: calcium-sensing receptor
ccRCC: clear cell RCC
ECM: extracellular matrix
ERK: extracellular signal-regulated kinase
FAK: focal adhesion kinase
JNK: c-Jun N-terminal kinase
MAPK: mitogen activated protein kinase
PI3K: phosphatidylinositol-4,5-bisphosphate 3-kinase
PSA: prostate specific antigen
RANK: receptor activator of NF-κB
RANKL: RANK ligand
RCC: renal cell carcinoma
SHC: SRC homology 2 containing-protein
