# Supplementary material for: Calcium-sensing receptor (CaSR) promotes development of bone metastasis in renal cell carcinoma
Source: Oncotarget. 2018 Mar 2;9(21):15766–79. doi: 10.18632/oncotarget.24607 (PMC5884663; doi:10.18632/oncotarget.24607)
Supplement: Supplementary file 1 [file oncotarget-09-15766-s001.pdf]

Calcium-sensing receptor (CaSR) promotes development of bone metastasis in renal cell carcinoma

SUPPLEMENTARY MATERIALS

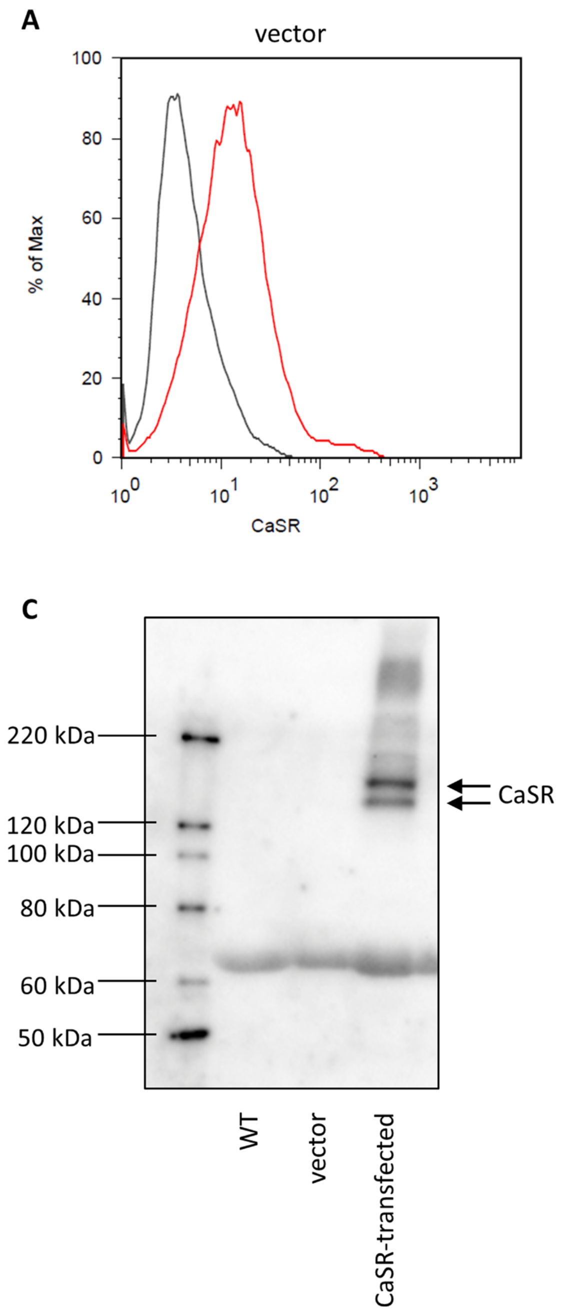

Supplementary Figure 1: Protein expression level of CaSR. The expression of CaSR was verified in transfected 786-O cells by flow cytometry (**A, B**) and Western blot analysis (**C**). Histogram of vector-transfected (A) and CaSR-transfected (B) 786-O cells (gray: isotype control, red: CaSR) are shown in percentage of maximum counts (relative cell number). Western blot of CaSR-transfected cells show the immature and the glycosylated form of CaSR (C).

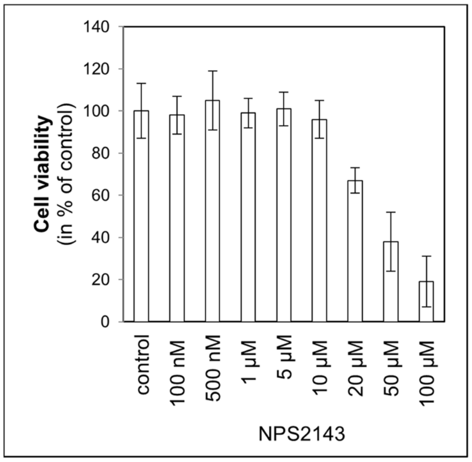

Supplementary Figure 2: Cell viability after treatment with NPS2143. The cell line 786-O was treated with different concentrations of NPS2143 and cell viability was determined with MTT. The cell vitality is shown as percentage of the vitality of untreated cells.

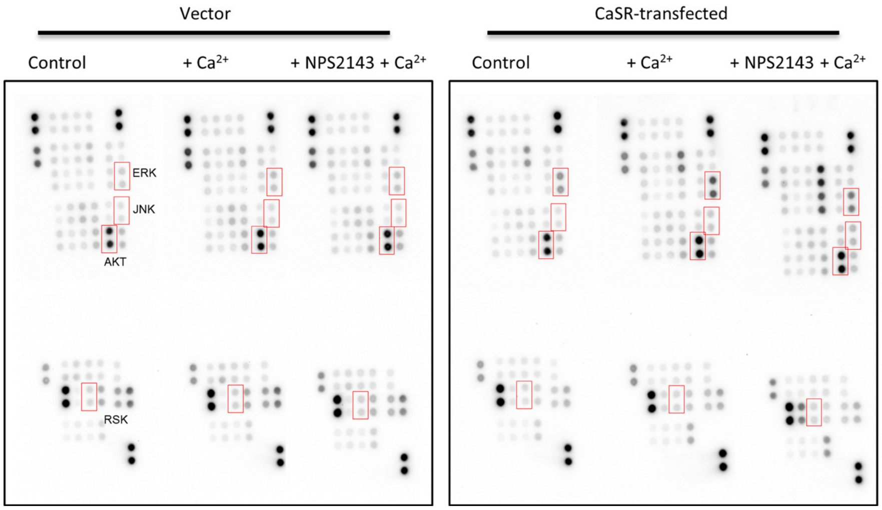

Supplementary Figure 3: Human phospho-kinase array of transfected cells. Cells were treated with calcium (5 mM) or a combination of calcium (5 mM) and NPS2143 (10 µM). Calcium triggered activity of AKT, JNK, ERK1/2 and RSK in CaSR-transfected cells.
